# Comprehensive exploration of geometric effects in sonoreactors: An extensive comparison of KI dosimetry, luminol maps and calorimetric power

**DOI:** 10.1016/j.ultsonch.2025.107395

**Published:** 2025-06-09

**Authors:** Igor Garcia-Vargas, Olivier Louisnard, Laurie Barthe

**Affiliations:** a Centre RAPSODEE, IMT Mines-Albi, UMR CNRS 5302, Université de Toulouse, 81013, Albi CT, France; b Laboratoire de Genie Chimique, Université de Toulouse, CNRS, INPT, UPS, Toulouse, France; c SinapTec, 7, Avenue Pierre et Marie Curie, 59260, Lezennes, France

**Keywords:** Ultrasound, Acoustic cavitation, Sono-chemiluminescence, Weissler reaction, Calorimetry

## Abstract

This study investigates sonochemistry results of Weissler reaction using a 20 kHz transducer immersed in three different glass vessels, under a total of 65 experimental geometrical configurations, already characterized in our previous papers through electrical, calorimetric and luminol measurements, and numerical simulation. In the first set of experiments, the transducer immersion depths is varied at constant liquid volume whereas in the second, the liquid level is altered at constant immersion. The study aims to identify the geometric conditions that maximize reaction rate, as measured by the concentration of triiodide ions  obtained after a given reaction time, and the overall sonochemical efficiency  , measuring the numbers of moles produced per joule dissipated in the liquid. By incorporating insights from the comparison between sono-chemiluminescence (SCL) of luminol and  dosimetry, this study establishes connections between global sonochemical effects and luminol mapping. For the three vessels considered, both indicators  and  evolve non-monotonically in function of either immersion or liquid level, and exhibit extrema for some specific configurations. No definite correlation could be established between maximal sonochemical effects and the corresponding shape and size of the luminol bright zones. Furthermore, the production of  does not correlate well with the volumetric energy density, suggesting that a fraction of the cavitation bubbles may heat the liquid without producing noticeable sonochemistry.

## Highlights

• The geometry of sonoreactors strongly influence Weissler reaction yield.
• The transducer immersion depth may change reaction rate by a 3-fold factor.
• Reaction yields are maximal for some intermediate immersion depths/liquid levels.
• There is no correlation between extension of luminol bright zones and Weissler reaction rates.
• Reaction rate is not correlated with dissipated energy.

## 1 Introduction

The use of large amplitude ultrasonic waves in liquids creates localized clouds of micro-bubbles filled with a gas/vapor mixture undergoing radial oscillations, a phenomenon termed as acoustic cavitation [1], [2], [3]. Above some threshold level of the acoustic source, these bubbles expand and further collapse on each oscillation cycle, generating localized high pressures and temperatures, with remarkable mechanical and thermal consequences [4]. Thermal energy concentration in these bubbles also leads to the formation of radicals, that can initiate and accelerate a series of chemical reactions [5], [6], [7]. This constitutes the basis of sonochemistry which has known a rapid growth over the last three decades, with numerous applications in organic synthesis [8], [9], [10], [11], [12], [13], [14], environmental remediation [15], [16], [17], [18], food processing [19], [20], [21], [22], [23] and many other fields in chemical engineering [24], [25], [26], [27]. Sonochemistry is a powerful method for promoting reactions that are difficult to achieve using conventional methods [28].

Although sonochemistry has many interesting applications, so far, ultrasound-enhanced chemical reactors have been used mainly on a laboratory scale [29]. The major challenges limiting their extrapolation to chemical engineering are to increase the energy efficiency of sonochemical processes and scale-up reactors for plant operational applications. The difficulty for that lies in the large variety and complexity of the nonlinear physical phenomena involved in acoustic cavitation, making the active bubble field non homogeneous in space and difficult to predict [30], [31], [32]. Moreover, the large variety of temporal and spatial scales prohibits so far a reasonable use of dimensional analysis, which is usually common in chemical engineering. Furthermore, there is no ready-to-use simulation code yet, despite large advances in modeling have been made recently [33], [34]. Within the large parameter set influencing the efficiency of ultrasonic reactors [28], [35], geometric factors have gained increasing attention [36], [37], [38]. Even for a single transducer immersed in a cylindrical vessel, there is yet no definite strategy to design the optimal vessel shape, liquid height and transducer immersion depth. Experimental characterization remain therefore an essential tool in the design of ultrasonic reactors.

Oxidation of the iodide ion (Weissler reaction) in aqueous solutions exposed to a continuous acoustic field was the first indication of the phenomena of homolytic breakdown of water in sonochemistry [39]. Since then, the use of KI dosimetry has been frequently used to describe the global chemical activity of ultrasound [40], [41], [42], both because it is straightforward and because it is a very trustworthy method. Its high sensitivity, simplicity, versatility, and non-invasive nature make it an ideal technique for studying the effects of ultrasound waves on chemical reactions in a variety of applications [7]. On the other hand, the precise assessment of the zones activated by acoustic cavitation remains difficult. In that respect, sono-chemiluminescence (SCL) constitutes an elegant, visual, and non-intrusive tool. It is based on the blue light emission of luminol as it is oxidized by the  radicals resulting from the water molecule cleavage in cavitation bubbles [43]. Both methods, KI dosimetry and SCL, complement each other and are frequently employed in tandem. The former provides a global estimation of the sonoreactor efficiency, while the latter allows to assess the localization of the activated zones.

The exploration of sonochemical processes has been a subject of intensive research, with recent studies shedding light on the intricate relationship between geometric parameters and the outcomes of ultrasonic systems. Renaudin et al. [44] explored the effects of liquid height on sonochemical luminescence intensity at a high frequency (500 kHz), revealing a diminishing impact as liquid height increased. Lim and co-workers [36] studied the oxidation of iodide ions using a 291 kHz sonicator with different volumes and showed that as the liquid height and ultrasonic power changed, the power density varied greatly and maximum cavitation yield of triiodide ions could be achieved for certain conditions. Similarly, Asakura et al. [29] evaluated the efficiency of a cylindrical sonoreactor as a function of frequency and liquid height using the KI dosimetry technique. They demonstrated that the latter two factors strongly influence the sonochemical efficiency. More recently, Son and colleagues [45] investigated geometry-based improvement of sonochemical activity, using a 20 kHz probe-type sonicator immersed in a 500 ml glass vessel. They studied the influence of geometric and operational factors, such as probe immersion depth, input power, liquid height, horizontal position of the probe and thickness of the support plate under the vessel. The optimal production rate of  production rate was obtained by the authors when the probe was placed near the bottom of the tank. Lee and co-workers [46] delved into the influence of liquid re-circulation. Their exploration covered a range of liquid heights, spanning 1 to 4 wavelengths, and varied flow rates from 1.5 to 6.0 L min^−1^. The collective findings from these studies contribute significantly to our understanding of the complex interplay between geometric parameters and sonochemical outcomes. These insights are crucial for refining the design and operation of ultrasonic systems across diverse experimental configurations.

The current investigation reports KI dosimetry measurements to explore the cavitational activity induced by a 20 kHz transducer immersed in three distinct glass vessels, systematically varying the geometrical conditions such as liquid height and transducer immersion depth. It complements earlier studies by our groups in which the same experimental configurations were analyzed by monitoring electrical data [47], calorimetry and luminol SCL [48], and numerical simulation [47], [48]. Different indicators are defined to estimate the efficiency of a given geometric configuration, either focusing on the rough chemical production, or accounting for the energy consumption. In order to address the reactors scale-up issue, a universal geometry-independent correlation between the latter indicators and various experimental inputs is sought. Finally, the present study also aims to establish connections between KI dosimetry, which measures global sonochemical effects and the corresponding luminol mapping, which unveils spatial information about radical production zones. Through this integrated approach, we seek a deeper understanding of the interplay between experimental parameters and cavitational outcomes.

## 2 Material and methods

### 2.1 Chemicals

Potassium iodide (99%), sulfuric acid 2 M, and sodium hydroxide (>97%) from Fisher Chemicals, as well as Luminol (3-aminophthalhydrazide, 98%) from Alfa Aesar, were used as received without additional purification. All solutions were prepared from distilled water at ambient temperature.

### 2.2 Experimental setup

**Fig. 1:**
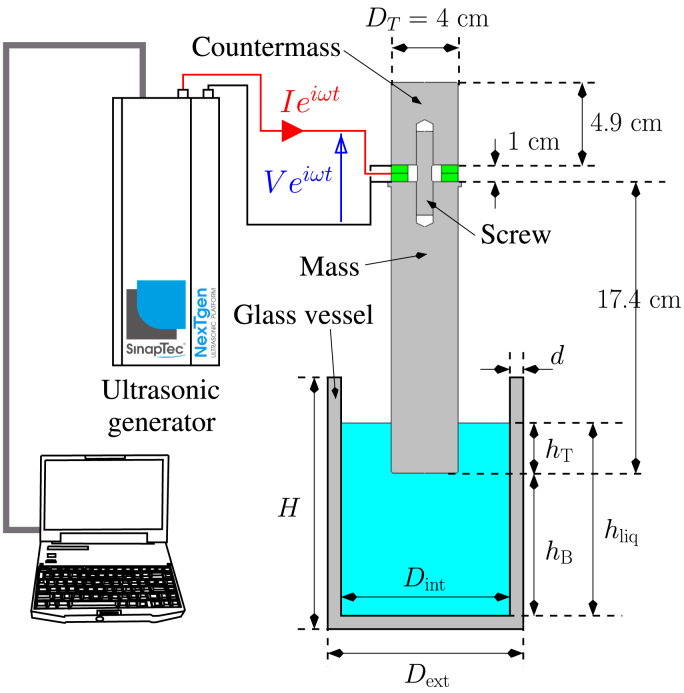
Schematic of the experimental setup.

The experimental setup is identical to that described in a previous work [47], [48], and is illustrated schematically in Fig. 1.

The transducer was placed in the center of three different commercial glass vessels, denoted as A, B, and C, which were filled with distilled water. These vessels have different volumetric capacities and geometries: vessel A has a capacity of 1 L and a wide form, vessel B has a capacity of 2 L and a narrow form, while vessel C has a capacity of 2 L and a wide form. The dimensions of the three vessels are reported in Table 1.

A 20 kHz homemade standard Langevin-type transducer (SinapTec, Lezennes, France) with two piezoelectric rings pre-stressed by a steel bolt between a mass and counter-mass constructed of titanium alloy was employed. The diameter of the emitting part is $D_{T}=\mathrm{40}\;\mathrm{mm}$. The specific resonance frequency of the unloaded transducer, $f_{0}=\mathrm{20 342}\;\mathrm{Hz}$, was measured using an impedance bridge. The transducer was driven by a computer-controlled ultrasonic generator (SinapTec NexTgen Inside 500), which allowed monitoring and logging of various electrical quantities every 50 ms, such as power, frequency, voltage, and current, as well as their mutual phase and impedance, during experiments. The transducer characteristics can be found in Ref. [47].

**Table 1:** Geometrical characteristics of the beakers (see Fig. 1).

Vessel	Form	Capacity (L )	$D_{\text{ext}}$ (cm )	H (cm )	d (cm )
A	Wide	1	10.56	14.3	2.3
B	Narrow	2	11.95	23.7	2.5
C	Wide	2	13.2	18.3	2.2

Two geometrical parameters can be varied (Fig. 1):

• the distance $h_{\text{B}}$ between the transducer tip and the vessel bottom,
• the water level $h_{\text{liq}}$.

The transducer immersion depth can also be obtained by:

$$h_{\text{T}}=h_{\text{liq}}-h_{\text{B}}.$$

In this study, two sets of experiments were carried out. In the first one, for each vessel, the input current of the transducer was fixed at a constant value of $I_{0,\text{RMS}}=\mathrm{0.6}\;A$ and $h_{\text{B}}$ was swept in a beaker-dependent range (Table 2) by changing the transducer immersion depth. The liquid volume $V_{\text{liq}}$ was kept constant so that the liquid level $h_{\text{liq}}$ varied in function of immersion depth $h_{\text{T}}$ following:

(1) $$V_{\text{liq}}=\pi \frac{D_{\text{int}}^{2}}{4}h_{\text{liq}}-\pi \frac{D_{T}^{2}}{4}h_{\text{T}}$$

where $D_{\text{int}}$ is the vessel internal diameter and $D_{T}$ is the transducer diameter. In the second set, the liquid height was varied and the position of the transducer inside the vessel was fixed at $h_{\text{B}}=\mathrm{20},\;\mathrm{80},\;\text{and}\mathrm{30}\;\mathrm{mm}$ for the vessel A, B and C, respectively, to allow a larger range for sweeping $h_{\text{liq}}$ in each case. Table 2 summarize the values of all parameters for the three series of experiments.

For each experiment, the liquid was irradiated continuously at ambient temperature and pressure. As the generator internal control loop was able to set the frequency in less than 1s, all the electrical data monitored during the first two seconds were removed. The insonification duration was 5 min for experiment set 1, and 3 min for experiment set 2. All tests and measurements were repeated three times, and the mean values and their respective standard deviations are reported in this study.

**Table 2:** Summary of geometric parameters used in experiments.

Experiment set	Vessel	$h_{\text{B}}$ (mm )	$h_{\text{liq}}$ (mm )	V (L )	$I_{0,\text{RMS}}$ A
Set 1	A	20 to 100	Computed from Eq. (1)	0.843	0.6
	B	80 to 170		1.835	
	C	30 to 130		1.767	

Set 2	A	20	30 to 120	Computed from Eq. (1)	0.6
	B	80	90 to 200		
	C	30	40 to 160		

### 2.3 Power measurement

The ultrasonic power dissipated in the liquid was determined experimentally by the calorimetric method, by measuring the temperature increase with time T(t), and using:

(2) $$P_{\text{cal}}=MC_{p}\frac{dT}{dt},$$

where $P_{\text{cal}}$ is the calorimetric power, $C_{p}$ = 4184 J kg^−1^ K^−1^ is the specific heat capacity of the liquid and M is the mass of liquid irradiated [49], [50].

The liquid temperature was recorded using a PT100 immersed directly into the reaction medium. The probe was consistently placed midway between the transducer lateral area and the vessel wall. The tip of the temperature probe was located about 40 mm above the vessel bottom when the liquid level was sufficiently high. For lower liquid levels, the probe tip was located roughly midway between the vessel bottom and the free surface.

To determine calorimetric power $P_{\text{cal}}$, we extracted the initial slope of the temperature vs. time curve during the first 2.5 min of sonication, where the curve is linear and heat losses can be safely neglected. This method is commonly employed in calorimetry to minimize errors due to thermal exchange with the environment.

A potential issue with the latter method is that owing to the inherent spatial inhomogenity of the bubble field, the associated heat source term and the resulting temperature field may be not be spatially uniform. This questions the sensitivity of the method to the precise location of the temperature probe. Section 2 in supplementary material discusses this point more deeply and shows that the measurement can be considered independent of the probe position.

### 2.4 Quantification of chemical activity

The sonochemical activity was assessed through two methods: the iodide method and luminol visualization. In the iodide method, a KI solution with a concentration of 10 g l^−1^ was employed.

To adjust and stabilize the pH between 4 and 7 and prevent reaction (11), small aliquots (100 µl) of concentrated sulfuric acid were added [40]. Despite sulfuric acid being a strong acid, this controlled dosing ensured a stable pH environment (near 5) without significantly altering the chemical composition of the system.

The primary reactions considered are [51]:

(3)
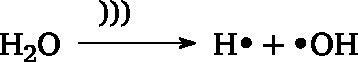

(4)
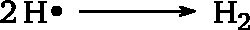

(5)
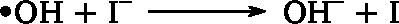

(6)
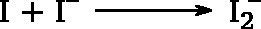

(7)
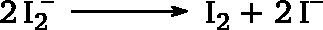

(8)
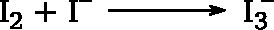

The iodide ions  , resulting from the ionization of KI dissolved in water, is easily oxidized by  radicals, hydrogen peroxide, etc, which are generated when water is irradiated with ultrasound to form iodine  , according to reactions (3)–(7). The triiodide ion (  ) is produced when excess  in solution combines with  (reaction (8)). The hydrogen atoms can then reduce the iodine formed as follows: (9)
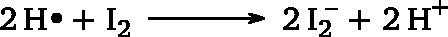
Therefore, relatively little iodine is produced in radiation chemistry. However, the reaction is less intense in sonochemistry because the amount of  generated is significantly lower than that of  [52]. Different reactions may take place depending on the type of solution. If the medium is acidic, the iodide can be oxidized by dissolved oxygen in solution [40]: (10)
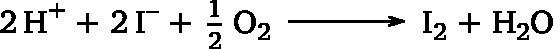
In a basic solution, however, hydrogen peroxide can react with triiodide: (11)
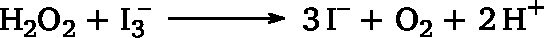
Iodide can also very slowly react with hydrogen peroxide as a result of hydroxyl radical recombination [53]: (12)
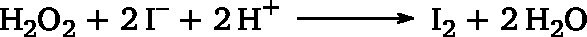
KI dosimetry is therefore an indirect method that quantifies the sonochemical activity in the reactor, mostly through the generation of  radicals during the implosion of active cavitation bubbles caused by the oxidation of iodide to triiodide. Using Beer Lambert’s equation, the  produced is measured by visible spectro-photometry at the highest absorption wavelength of 350 nm:

(13) $$A=\epsilon_{\lambda }\;\ell \;C,$$

where $\epsilon_{\lambda }$ is the molar extinction factor (ϵ_350 nm_ = 26 303 L mol^−1^ cm^−1^), C the concentration of the compound, and ℓ the optical path length (ℓ=1cm). The *sonochemical efficiency* SE can be defined as the number of moles of triiodide formed per unit of ultrasonic energy dissipated in the liquid, in order to assess the energetic efficiency of the sonochemical oxidation [41], [45], [54], [55]: (14)
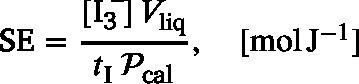
where  is the concentration of  produced, $V_{\mathrm{liq}}$ is the liquid volume, $t_{I}$ is the ultrasound irradiation time, and $P_{\text{cal}}$ is the calorimetric power.

Luminol excitation was used to visualize cavitational activity in the vessels. The experimental approach closely follows the methodology detailed in our prior publication [48], and is recalled in Supplementary material (Sec. S1).

## 3 Results and discussion

### 3.1 Effect of the transducer immersion

To investigate the influence of transducer immersion on cavitational activity, a series of experiments were conducted using a 20 kHz transducer immersed in three different glass vessels. The transducer was positioned at different depths within each vessel, ranging from near the bottom to a higher position closer to the liquid surface. Calorimetry, KI dosimetry and SCL techniques were utilized to assess the influence of this geometric parameter.

**Fig. 2:**
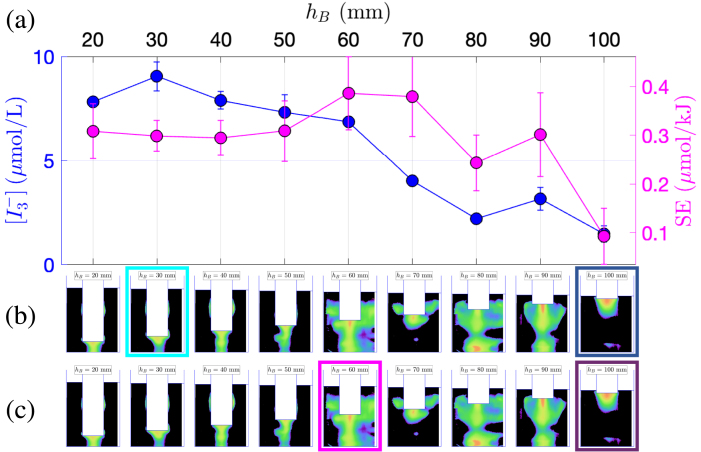
Comparison between KI dosimetry results and luminol maps for experiments in the 1 L beaker (vessel A) with varying immersion depth. (a)  production (blue curve, left y-axis) and SE (pink curve, right y-axis) in function of $h_{\text{B}}$. (b) Corresponding luminol maps. The map corresponding to maximum  is highlighted in light blue, and the one corresponding to minimum  is highlighted in dark blue. (c) Corresponding luminol maps. The map corresponding to maximum SE is highlighted in pink, the one corresponding to minimum SE is highlighted in violet.

**Fig. 3:**
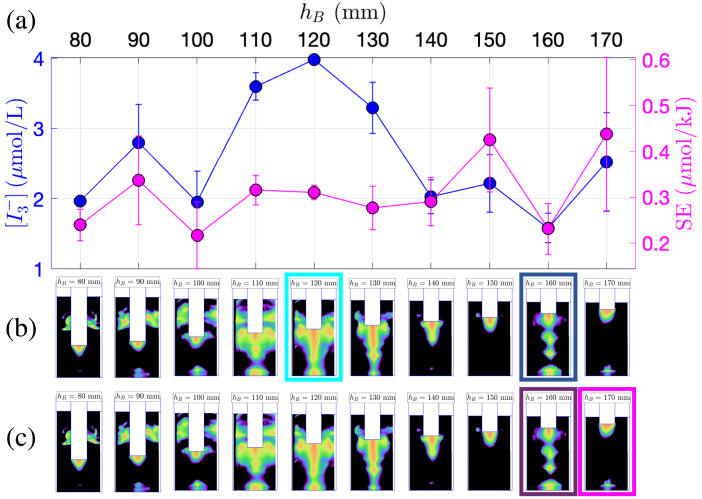
Comparison between KI dosimetry results and luminol maps for experiments in the narrow 2 L beaker (vessel B) with varying immersion depth. (a)  production (blue curve, left y-axis) and SE (pink curve, right y-axis) in function of $h_{\text{B}}$. (b) Corresponding luminol maps. The map corresponding to maximum  is highlighted in light blue, and the one corresponding to minimum  is highlighted in dark blue. (c) Corresponding luminol maps. The map corresponding to maximum SE is highlighted in pink, the one corresponding to minimum SE is highlighted in violet.

**Fig. 4:**
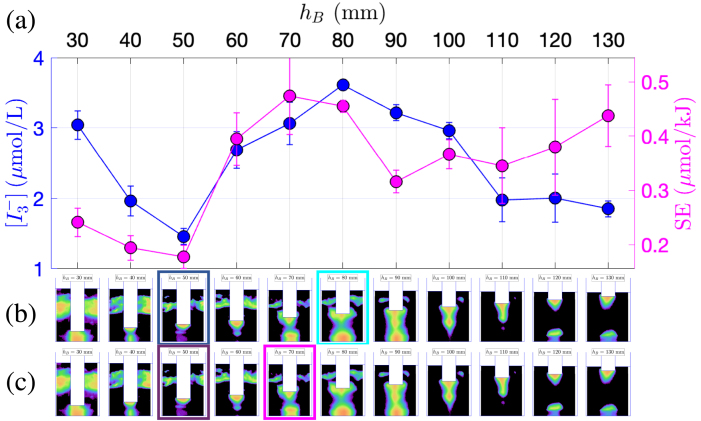
Comparison between KI dosimetry results and luminol maps for experiments in the wide 2 L beaker (vessel C) with varying immersion depth. (a)  production (blue curve, left y-axis) and SE (pink curve, right y-axis) in function of $h_{\text{B}}$. (b) Corresponding luminol maps. The map corresponding to maximum  is highlighted in light blue, and the one corresponding to minimum  is highlighted in dark blue. (c) Corresponding luminol maps. The map corresponding to maximum SE is highlighted in pink, the one corresponding to minimum SE is highlighted in violet.

Fig. 2a presents the dosimetry for the 1 L beaker, illustrating the production of triiodide concentration  (blue curve, left ordinate axis), and the sonochemical efficiency SE (pink curve, right ordinate axis). It should be noted that on this figure, the transducer immersion depth increases from right to left. Interestingly, the maxima of the two quantities do not correspond to the same immersion depths: the maximum triiodide concentration is observed at $h_{\text{B}}=\mathrm{30}\;\mathrm{mm}$, whereas the maximum sonochemical efficiency occurs at $h_{\text{B}}=\mathrm{60}\;\mathrm{mm}$. This conclusion also holds for vessels B and C (Figs. 3a and 4a, respectively). Previous works investigating geometric effects also suggested that transducer immersion depth significantly influences the cavitational activity. Son and co-workers [45] showed that a deeper immersion of the transducer within the liquid medium resulted in enhanced cavitation, as evidenced by both calorimetric measurements and  production rate determined through KI dosimetry. The authors claimed that this observation can be attributed to the increased proximity of the transducer to the liquid bulk, leading to a more efficient transfer of ultrasonic energy. Albeit the latter argument is intuitively satisfactory, the present results show that this interpretation should be qualified, and that geometry effects are more involved. Indeed, whereas the latter rule is approximately fulfilled for vessel A (Fig. 2a), it is no longer true for vessels B and C (Figs. 3a and 4a) where non-monotonic curves in function of immersion can be clearly observed.

The three sets of experiments also evidence that the optimal immersion depth is not the same depending on whether one seeks a maximal sonochemical production or a maximal yield for the minimum energy consumption. For example in vessel C, when the transducer is located close to the bottom of the vessel (leftmost points of the curves), a high concentration is obtained along with low efficiency SE, which indicates a high production but at the price of a large energetic cost. The opposite holds for the last point of the curve ($h_{\text{B}}=\mathrm{130}\;\mathrm{mm}$), with the transducer positioned farther from the bottom.

The minima for  and SE are attained at the same immersion depth and this property was found to hold for the three vessels. Interestingly, these minima do not necessarily correspond to small immersion depths, especially in the case of vessel C (Fig. 4a), where the minimum of both quantities is obtained for $h_{\text{B}}=\mathrm{50}\;\mathrm{mm}$, a relatively high immersion depth. Why this specific geometric configuration is unfavorable for sonochemistry remains to be explained and probably originates from acoustic effects. More intriguing, this worst configuration is somewhat close to the optimal ones, and a sharp increase of both  and SE can be observed in the range $h_{\text{B}}\in \left[\mathrm{50}\;\mathrm{mm},\mathrm{80}\;\mathrm{mm}\right]$. More precisely, it can be seen that for vessel C at constant generator position and constant liquid volume, any slight change in the transducer immersion depth can increase or decrease production by more than a two-fold factor.

More information may be gathered from the corresponding luminol maps. Fig. 2b and c also display the luminol maps obtained in the same geometric conditions as the KI experiments. The images series is repeated twice: in Fig. 2b, the maps associated to the maximum  production is framed in light blue, and the minimum  in dark blue. Similarly, in Fig. 2c, the pink frame denotes a maximal value of SE and the violet frame a minimal one. The same presentation is adopted for vessel B (Fig. 3b, c) and vessel C (Fig. 4b, c).

For vessel A, upon examination of Fig. 2b, the luminol image corresponding to the maximum  (for $h_{\text{B}}=\mathrm{30}\;\mathrm{mm}$) does not exhibit any specific shape, apart from a conical structure under the transducer, and the presence of very thin lateral structures. Conversely, Fig. 2c reveals that maximal SE (for $h_{\text{B}}=\mathrm{60}\;\mathrm{mm}$) corresponds to luminol zones invading almost all the vessel, with large lateral structures. For vessel B, Fig. 3b reveals that the maximum  is observed (for $h_{\text{B}}=\mathrm{120}\;\mathrm{mm}$) when an “elongated T”-shaped structure is formed under the transducer, accompanied by the presence of lateral structures. This shapes were conjectured to originate from streaming, and the latter is known to originate from a strong acoustic field yielding strong attenuation under the transducer [56]. Comparatively, the luminol map corresponding to the maximum SE (at $h_{\text{B}}=\mathrm{150}\;\mathrm{mm}$, pink frame on Fig. 3c) has a very simple shape with an activity spot located under the transducer. Interestingly, this immersion depth corresponds to a rather low value of  , but  remains large here because for this immersion depth, $P_{\text{cal}}$ is very low [48]. The luminol patterns in vessel C (Fig. 4b, c) reveal a progressive disappearance of lateral bright zones as immersion depth is decreased. Secondary bright zones near the vessel bottom also appear and disappear in given ranges of immersion depths. Maximal  and SE occur very closely and seem to match an intense zone under the transducer tip, along with both extinguishing lateral zones and a very bright bottom zone. The appearance and disappearance of the latter seems moreover to be correlated with the sharp variations of  in the interval [50mm,110mm].

The luminol patterns associated with the coinciding minima of  and SE display distinct features for the three vessels. For vessel A (Fig. 2b, c), the luminol bright zone is restricted to a large hemispherical spot attached to the transducer (which is barely immersed in the liquid). For vessel B (Fig. 3b, c), the minimum is concomitant with roughly equidistant secondary bright spots located along the axis of symmetry, reminiscent of a standing wave pattern (at $h_{\text{B}}=\mathrm{160}\;\mathrm{mm}$). For vessel C (Fig. 4b, c), lateral structures are still largely developed, even more than those observed than for maximal production conditions. This might suggest that such lateral structure have a modest effect on the sonochemical yield.

In summary, for the experimental set at constant liquid volume with a variable immersion depths, no definite qualitative correlation between the shape of luminol maps and extremal  or SE seems to be uniformly valid for the three vessels.

### 3.2 Effect of the liquid height

**Fig. 5:**
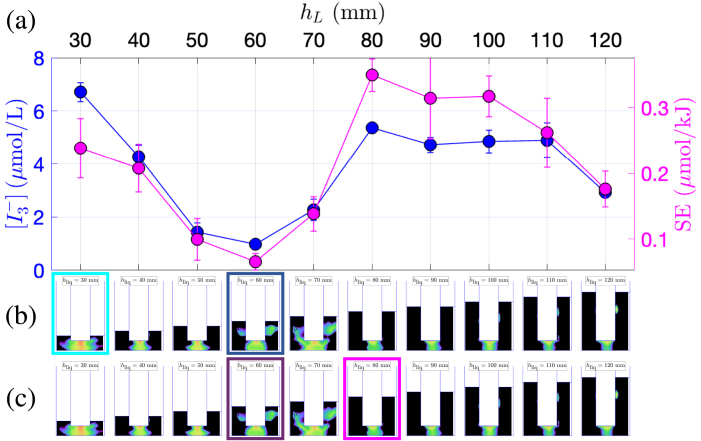
Comparison between KI dosimetry results and luminol maps for experiments in the 1 L beaker (vessel A) with varying liquid level. (a)  production (blue curve, left y-axis) and SE (pink curve, right y-axis) in function of $h_{\text{liq}}$. (b) Corresponding luminol maps. The map corresponding to maximum  is highlighted in light blue, and the one corresponding to minimum  is highlighted in dark blue. (c) Corresponding luminol maps. The map corresponding to maximum SE is highlighted in pink, the one corresponding to minimum SE is highlighted in violet.

**Fig. 6:**
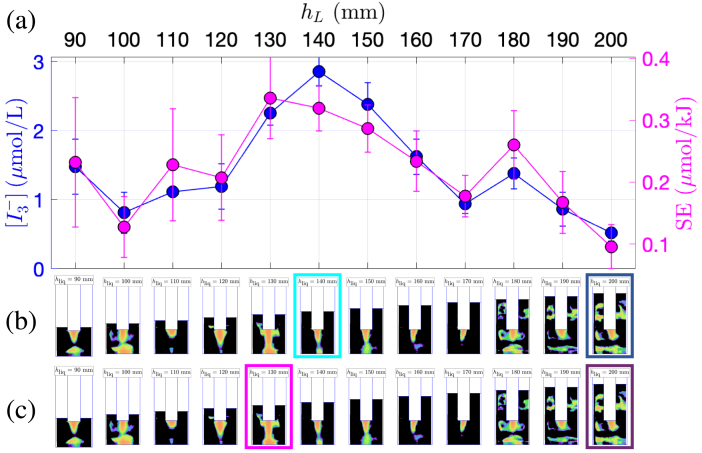
Comparison between KI dosimetry results and luminol maps for experiments in the narrow 2 L beaker (vessel B) with varying liquid level. (a)  production (blue curve, left y-axis) and SE (pink curve, right y-axis) in function of $h_{\text{liq}}$. (b) Corresponding luminol maps. The map corresponding to maximum  is highlighted in light blue, and the one corresponding to minimum  is highlighted in dark blue. (c) Corresponding luminol maps. The map corresponding to maximum SE is highlighted in pink, the one corresponding to minimum SE is highlighted in violet.

**Fig. 7:**
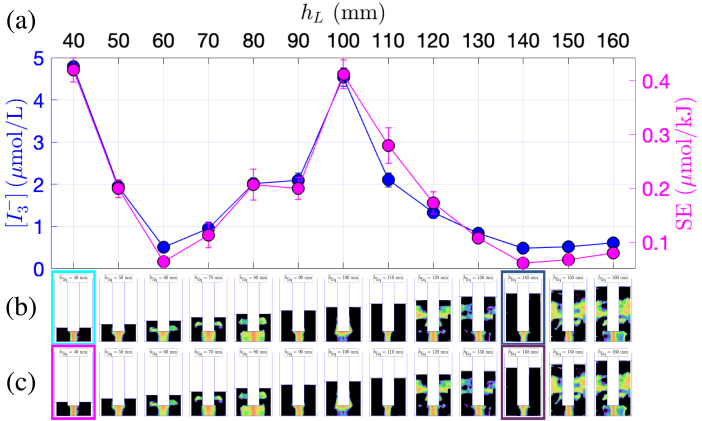
Comparison between KI dosimetry results and luminol maps for experiments in the wide 2 L beaker (vessel C) with varying liquid level. (a)  production (blue curve, left y-axis) and SE (pink curve, right y-axis) in function of $h_{\text{liq}}$. (b) Corresponding luminol maps. The map corresponding to maximum  is highlighted in light blue, and the one corresponding to minimum  is highlighted in dark blue. (c) Corresponding luminol maps. The map corresponding to maximum SE is highlighted in pink, the one corresponding to minimum SE is highlighted in violet.

The impact of liquid height was investigated following the same procedure and adopting a similar presentation for the results (Figs. 5, 6 and 7 for vessels A, B and C, respectively). It can be seen that the  curve and the SE curve have approximately the same shapes within each set of experiments. The minima of the two quantities are found for the same liquid level as for the experiments with varying immersion, but moreover, this is also almost true here for the maxima. For the three vessels, the curves are markedly non monotonic so that the maxima are obtained in each set for an intermediate liquid depth.

For vessel A, (Fig. 5b, c), luminol maps reveal that lateral structures seldom appear. Rather counter-intuitively, their appearance (at $h_{\text{liq}}=\mathrm{60}\;\mathrm{mm}$) matches a minimum  and SE. The latter remark also holds for the two other vessels, and again, this underlines the weak role of these lateral cavitation zone in global sonochemistry. Between $h_{\text{liq}}=\mathrm{80}\;\mathrm{mm}\;\text{and}\;\mathrm{120}\;\mathrm{mm}$, the topology of the luminol maps remains similar: a conical structure of constant dimensions is visible at the transducer tip accompanied by very thin lateral structures, corresponding to a nearly constant  and SE production.

Vessel B exhibits a similar trend with a global maximum of  at $h_{\text{liq}}=\mathrm{140}\;\mathrm{mm}$, the maximum of SE being very close. The corresponding luminol bright region has a jet-like shape, probably originating from acoustic streaming. Conversely, the lowest chemical yield (corresponding to the largest liquid volume, $h_{\text{liq}}=\mathrm{200}\;\mathrm{mm}$) exhibits a very rich luminol structure with a large spot under the transducer, well-developed lateral bright zones, not only near the transducer lateral area but also below, and a secondary structure near the vessel bottom. This sounds counter-intuitive again, but it can be noted that, as for other experiments, the minimum chemical yield matches luminol maps that are reminiscent of standing wave patterns. This is in contradiction with other results in the literature [55].

Lastly, examining the behavior in vessel C (Fig. 7) reveals similar conclusions. The largest yield is obtained at the lowest liquid level but interestingly, similarly large values can be obtained for intermediate filling levels ($h_{\text{B}}=\mathrm{100}\;\mathrm{mm}$). The luminol bright zone in the latter case takes an unusual wide shape, covering the whole transducer tip (Fig. 7b). Looking back to the precedent sets of experiments, this particular structure shares some similarities with the ones corresponding to maxima of  in Figs. 2b and 4b. It seems therefore to be strongly correlated with highly active cavitation, but its precise origin remains to be explained. Here again, maximum  and SE points are concomitant with the absence of lateral structures, and quite the contrary, the presence of the latter is always related to low values of both quantities. However, they are absent for the absolute minimum sonochemical yield at $h_{\text{liq}}=\mathrm{140}\;\mathrm{mm}$. Amazingly, it may be noted that for the latter, the luminol map does not exhibit some striking differences with the one corresponding to the maximum production at $h_{\text{liq}}=\mathrm{40}\;\mathrm{mm}$.

### 3.3 Energetic aspects

**Fig. 8:**
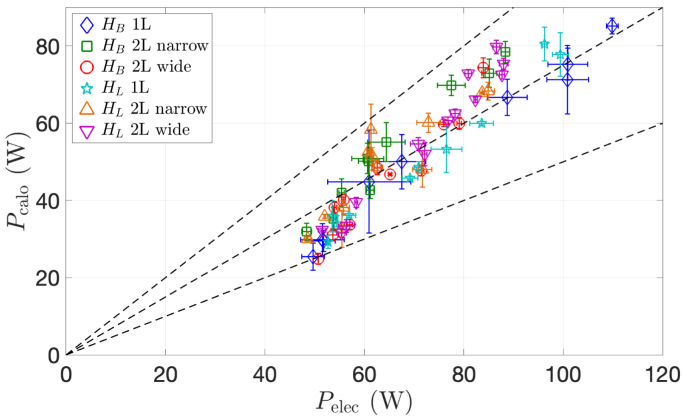
Calorimetric power $P_{\text{cal}}$ in function of electrical power $P_{\text{elec}}$ for all experiments. The dashed line represents $P_{\text{cal}}/P_{\text{elec}}=0.5$, 0.75 and 1(from bottom to top).

It is first instructive to compare calorimetric power and consumed electrical power. The ratio of the former to the latter is the transducer yield. Fig. 8 displays $P_{\text{cal}}$ in function of $P_{\text{elec}}$ for all experiments. The transducer yield ranges between 0.5 and 1 for all experiments, and better yields are obtained for large powers.

Then, in order to conduct a more comprehensive analysis of the sonochemical activity, we sought to examine globally the results of all sets of experiments by considering a parameter other than the geometric parameters $h_{\text{B}}$ and $h_{\text{liq}}$. A common approach in such cases is to introduce a quantity known as the *Energy Density* [55], [57], defined as follows:

(15) $$\text{Ev}=\frac{P_{\text{cal}}\;t_{I}}{V_{\mathrm{liq}}},\;\left[\text{J}\;\text{L}{}^{\mathrm{-1}}\right]$$

where $P_{\text{cal}}$ is the calorimetric power, $V_{\mathrm{liq}}$ is the solution volume and $t_{I}$ is the irradiation time. This quantity represents the mean energy dissipated in the liquid per unit volume.

**Fig. 9:**
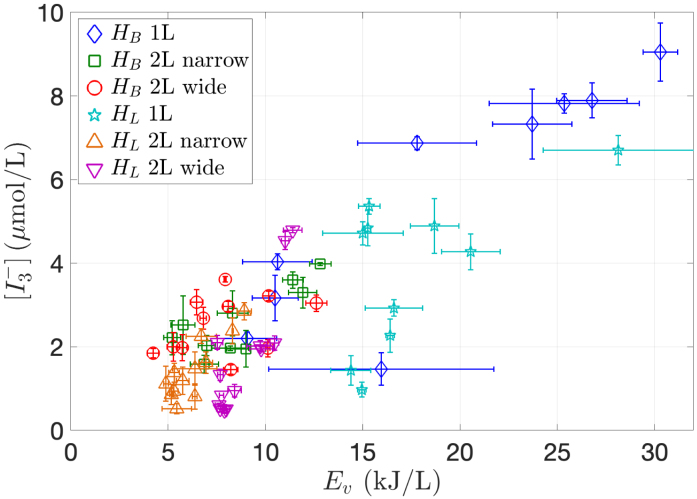
Concentration  produced in function of energy density (Ev) for all vessels. The input current was $I_{0,\text{RMS}}=\mathrm{0.6}\;A$ in all cases. The liquid height was computed from Eq. (1) for the experiments in set 1 and the distance between the transducer’s tip and the vessel’s bottom was 20 mm, 80 mm, and 30 mm for the vessels A, B and C, respectively, in set 2.

**Fig. 10:**
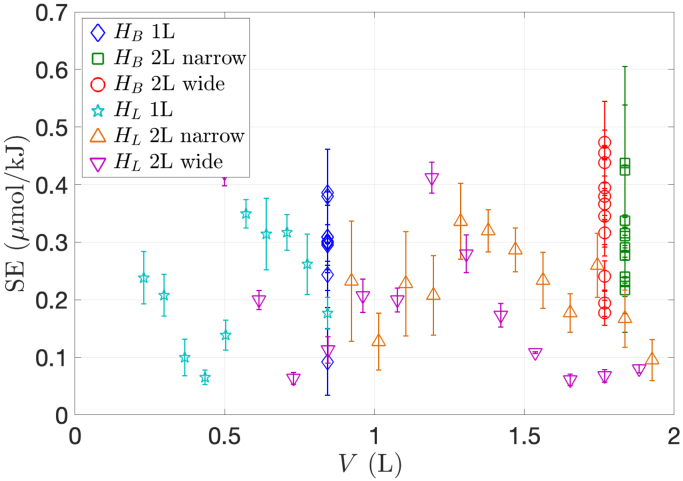
Sonochemical efficiency SE in function of liquid volume V for all sets of experiments. The input current was $I_{0,\text{RMS}}=\mathrm{0.6}\;A$ in all experiments.

Fig. 9 provides a comprehensive visualization of the  concentration plotted against the energy density $E_{v}$ for all experiments combined. The data points look very dispersed and no net correlation can be observed. This finding in opposition with the results of Merouani et al. [58], who found a linear dependence between the product concentration and energy density $E_{v}$ not only for KI oxidation, but also for other classical sonochemical reactions. Their result may sound rational and would indicate that the number of chemical bonds broken is directly proportional to the acoustic energy injected in the liquid. Since from the definitions,  , the consequence of this linear law is that SE should be volume independent, as could be actually checked by Merouani and co-workers on their experimental results. This cannot be the case for the present experiments as evidenced by the SE curves of Fig. 5, Fig. 6, Fig. 7 (pink lines), if ones recalls that the liquid volume is an affine function of liquid height. Plotting SE in function of volume for all experiments combined clearly confirms this conclusion (Fig. 10). This difference can be attributed to the distinct experimental conditions used: Merouani and co-workers [58] worked with a vessel insonified by a 300 kHz driven piezoelectric disk fixed on the bottom. This prohibits examination of immersion depth effects, lateral cavitation zones, contrarily to our experiments performed with an immersed transducer. Additionally, it may be conjectured that the higher frequency used by the authors and their emitter configuration is less subject to acoustic effects than in the present experiments.

Fig. 9 also exhibits data points that appear strongly deviating from the general trend, particularly for some experiments in the 1 L vessel with varying liquid level (light blue  symbols), and also for the lowest immersion depth experiment in the latter vessel (blue ◊ symbols). In fact, Fig. 10 shows that this dispersion is also large for the results at varying volume in the two 2 L vessels (purple ▽ symbols, and orange △ symbols) but it is less visible in the representation of Fig. 9 since the latter displays volumetric quantities.

The present results suggest therefore that directly correlating the quantity of a sonochemical product to the acoustic energy dissipated in the liquid is way far from justified, despite the idea sounds intuitive and some earlier results support this correlation [58]. This conclusion can be easily explained physically: the liquid heats under insonification by a variety of phenomena (liquid shearing and nearly adiabatic heating of the bubble interior [33], [59]). These phenomena become intense as soon as bubbles becomes inertial, roughly at the Blake threshold. But chemical production requires a further energy densification in the bubble core [60], [61], [62] so that the threshold for chemistry is larger than the inertial cavitation threshold. On the other hand, Mettin and co-workers could evidence, by slightly modifying the driving frequency or amplitude, that two classes of inertial bubbles could be observed in the same experimental setup: a spherical chemically inactive bubble, or clustering chemically active bubbles. The latter were suspected to undergo non-spherical collapses [63], despite no direct observation could directly support this conclusion. Thus, in a given experiment, acoustic effects may produce inertial chemically active bubbles in some zones, and inertial chemically inactive bubbles in others. Being oscillating inertially, both would contribute to energy dissipation, but not to sonochemistry. The ratio of both populations may change with geometry because of acoustic effects, so that within a set of experiments where geometry is varied, sonochemical production may be badly correlated with the acoustic energy dissipated in the liquid.

In summary, there exists a range of bubbles that can be thermally active but chemically inefficient. Furthermore, the threshold for chemical activity is most likely dependent on the chemical reaction considered, so that some bubbles may be more chemically active for some reactions than for others. This may explain, at least qualitatively, some of the surprising results obtained in the comparison between luminol maps and  production presented in Sections 3.1, 3.2. The question arises of the respective locations of chemically active and inactive inertial bubbles. From recent simulations accounting for bubble size poly-dispersity, there are hints that bubbles under the transducer and the ones in lateral zones do not have the same size [34].

Finally, the question remains why some geometrical configurations are more favorable than others. The explanation probably lies in acoustic effects and more information could be gathered if one could reconstruct pressure and velocity at the transducer tip, and therefore acoustic impedance seen by the latter, which can be deduced from the electrical voltage and current across the transducer. Work is in progress, and there are hints that optimal sonochemical configurations may correspond to a specific phase of the acoustic impedance [64].

## 4 Conclusions

This comprehensive study delved into the complexities of sonochemical efficiency and radical production within three vessels of distinct sizes and shapes, exploring a total of 65 geometric configurations, by varying either the transducer immersion depth or the liquid height. The Weissler reaction was performed in each configuration and the results were systematically compared to luminol images obtained in the same conditions. The nuanced findings present several noteworthy observations and implications, shedding light on the complex interplay between geometric parameters and sonochemical outcomes.

First and foremost, the results of our experiments with variable transducer immersion underscore a noticeable sensitivity of sonochemical production to the geometric configuration, even at constant liquid volume. In a previous study by our groups [47], it was already shown that the power dissipated in the liquid could vary in a large range even if the liquid volume and generator power graduation were kept constant. A pivotal result of the present study is that this is also true for the sonochemical outcome. In summary, two experimenters utilizing the same graduation level on the generator and same liquid volume, but different immersion depths, may observe sonochemical yields varying by up to a three-fold factor.

On the other hand, a similar evolution was found in all vessels for the sonochemical production and sonochemical efficiency when the liquid level was varied. The evolution is way far from monotonic, and both quantities reach minimum and maximum values respectively for some specific liquid levels. This also constitutes a crucial issue in connection with sonoreactors scale-up.

Luminol maps, despite their counter-intuitive nature, provided some insights into sonochemical performance. Configurations yielding maximum concentration and sonochemical efficiency of Weissler reaction often coincided with the absence of lateral structures, whereas the presence of the latter often correlated with lower sonochemical yields and sonochemical efficiencies. Additionally, some configurations associated with low sonochemical yields exhibited luminol maps reminiscent of standing wave patterns. A rather intriguing result is the absence of correlation between the spatial extension of the luminol glowing zones and the sonochemical yield. This suggests that geometric configurations exhibiting extended luminol bright zones are not necessary optimal for the sonochemical yield of another reaction.

The full set of results of the present experimental campaign was also combined with the calorimetric powers measured in the same conditions and published earlier [48]. Contrarily to other results reported in the literature, the quantity of matter produced by sonochemistry was found uncorrelated with the energy dissipated in the liquid. This suggests that some fraction of the inertial cavitation bubbles may heat the liquid without contributing significantly to sonochemistry, and this fraction is geometry-dependent.

Whereas our experiments demonstrate the strong influence of geometric parameters, there is a lack of theoretical explanation on its origin. Clearly, acoustic effects are at work but their detailed mechanisms remain elusive, and are probably complex owing to the presence of cavitation. Further investigation is warranted to understand the underlying physics contributing to this behavior. Reconstructing acoustic quantities (velocity and force) at the transducer emitting area would shed more light on these effects. Furthermore, modeling and simulation of acoustic and bubble fields has considerably evolved in the last decade and the present set of results constitute a substantial basis for testing future models. Finally, albeit consequent, the geometric parameter set scanned in this study should be enlarged and extended to larger volumes.

## CRediT authorship contribution statement

**Igor Garcia-Vargas:** Writing – original draft, Visualization, Validation, Software, Methodology, Investigation, Formal analysis, Data curation. **Olivier Louisnard:** Writing – review & editing, Visualization, Supervision, Software, Resources, Project administration, Investigation, Funding acquisition, Formal analysis, Data curation, Conceptualization. **Laurie Barthe:** Writing – review & editing, Validation, Supervision, Resources, Project administration, Methodology, Investigation, Funding acquisition, Formal analysis, Conceptualization.

## Declaration of competing interest

The authors declare the following financial interests/personal relationships which may be considered as potential competing interests: Olivier LOUISNARD reports financial support was provided by SinapTec. Laurie BARTHE reports financial support was provided by SinapTec. Igor GARCIA-VARGAS reports a relationship with SinapTec that includes: employment and funding grants.

## Appendix A Supplementary data

MMC S1 Detailed description of the luminol method (fundamentals, experimental conditions, image treatment) and of the calorimetric method (influence of the temperature probe location).
