# Supplementary material for: Comprehensive exploration of geometric effects in sonoreactors: An extensive comparison of KI dosimetry, luminol maps and calorimetric power
Source: Ultrason Sonochem. 2025 Jun 9;120:107395. doi: 10.1016/j.ultsonch.2025.107395 (PMC12206022; doi:10.1016/j.ultsonch.2025.107395)
Supplement: MMC S1 — Detailed description of the luminol method (fundamentals, experimental conditions, image treatment) and of the calorimetric method (influence of the temperature probe location). [file mmc1.pdf]

Supporting information

Comprehensive exploration of geometric effects in sonoreactors: an extensive comparison of KI dosimetry, luminol maps and calorimetric power.

Igor Garcia-Vargas[a,b,c], Olivier Louisnard[a,*], and Laurie Barthe[b]

[a]*Centre RAPSODEE, IMT Mines-Albi, UMR CNRS 5302, Université de Toulouse, 81013, Albi CT, France*
[b]*Laboratoire de Génie Chimique, Université de Toulouse, CNRS, INPT, UPS, Toulouse, France*
[c]*SinapTec, 7, Avenue Pierre et Marie Curie, 59260, Lezennes, France*

1 Luminol method

1.1 Chemistry

Luminol molecules, when exposed to ultrasonic irradiation, react with hydrogen peroxide and free radicals generated during water sonolysis, resulting in luminous 3-aminophthalate production and visible light emission. This emission is referred to as SCL, and it is illustrated in the schematic diagram displayed in Fig. 1 (after Ref. [1]).

Under experimental conditions characterized by a basic pH, the primary species is the luminol monoanion (I), as luminol is a weak dibasic acid with respective pKa values of 6.3 and approximately 13 for its first and second dissociation stages [2]. The initial step (i) illustrated in Figure 1 involves the oxidation of (I) by radicals such as •OH, yielding the diazaquinone radical anion (II). Subsequently, in step (ii), (II) interacts with O_2^-, resulting in the formation of the hydroperoxide adduct (III), which exhibits a pKa of 10.4, according to Lind et al. [3]. Notably, only the monoanion form of III decomposes in step (iii), giving rise to the excited state of the aminophthalate monoanion (I). Concurrently, the neutral form of (III) undergoes decomposition via a dark reaction (iv), leading to the regeneration of the initial reactive species (I) alongside O_2. It is postulated that step (iii) follows a concerted mechanism involving an unstable endoperoxide intermediate [3], with the aminophthalate product (IV) returning to its ground state, emitting light at a wavelength of 430 nm.

1.2 Experimental

In the present work, a solution composed of 0.1 g L^{-1} luminol (3-aminophthalhydrazide) and 1 g L^{-1} NaOH in distilled water [4–6]

Sonochemiluminescence images were captured using a digital exposure-controlled camera (Sony α 7 III with a Sony FE lens of $f = 50$ mm) in a light-sealed room. Each exposure lasted 20 seconds with an aperture set to $f/8$.

*olivier.louisnard@mines-albi.fr, Corresponding author

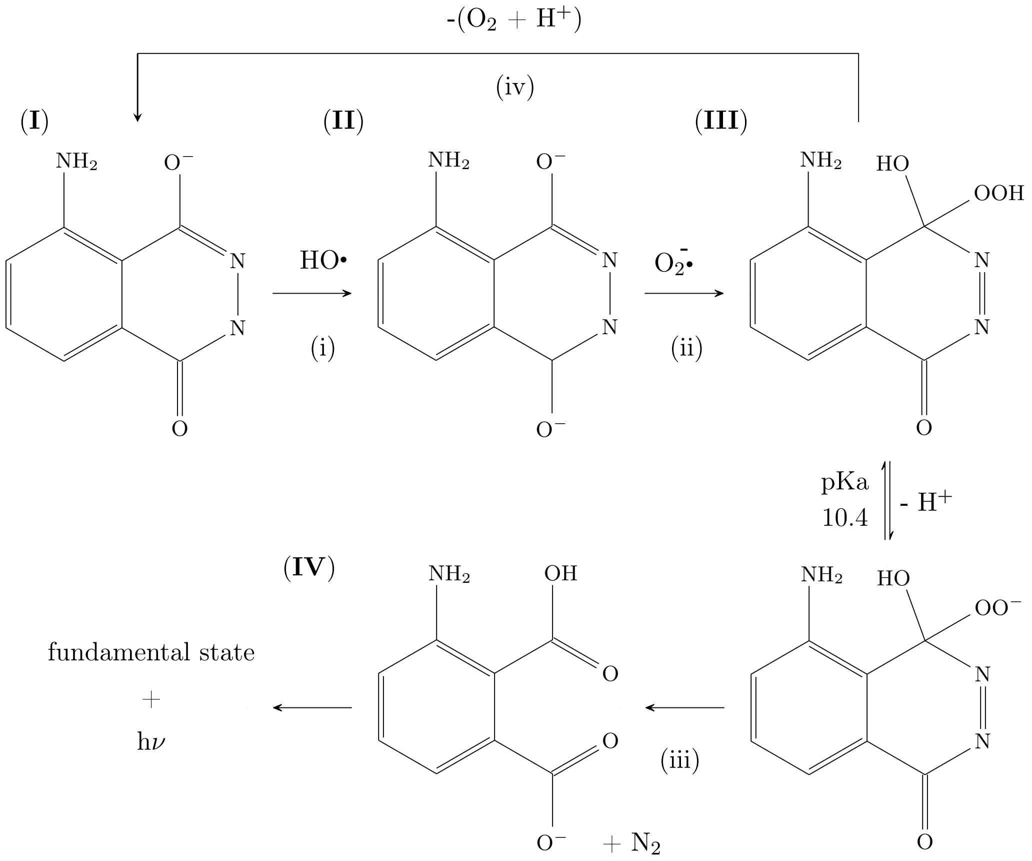

Figure 1: Reaction scheme of sono-chemiluminescence of luminol (from Ref. [1]).

1.3 Image treatment

To address distortions in cylindrical vessels, a Matlab-based post-processing algorithm was employed, correcting optical aberrations through a simplified ray-tracing method. Finally, a perceptual color map, chosen for clarity and accessibility, was adopted from Ref. [7], avoiding misinterpretation of spurious gradients associated with standard color maps, such as the classical rainbow one [8, 9]. This method ensures reliable and comparable sonochemiluminescence data, aligning with our commitment to precision and transparency in our investigation into the influence of geometric parameters on sonochemical outcomes.

It should be noted that the way we converted natural blue color emitted by luminol to false color slightly differs in the present paper compared to [6]. In the latter, the false colormap was mapped to the *minimum/maximum blue intensity range for each image.* As pointed out by a reviewer, this had the disadvantage that an orange color may correspond to a different intensity from image to image. To avoid this side-effect in the present presentation, we chose to map the false colormap to the *minimum/maximum blue intensity range over the full images of the current experiment.* In other words, a given color has the same meaning for all images of a given line in Figs 2-7.

2 Calorimetric method

Acoustic cavitation is inherently inhomogeneous, as evidenced experimentally in the present work by luminol maps. This may therefore yield non-homogeneous temperature fields within

the liquid, so that the slope of the time-temperature curve recorded by the probe may in fact measure a local volumetric power. The estimate of $\mathcal{P}_{\rm cal}$ may therefore depend on the localization of the probe. This is a potential drawback of the method, which is however currently used without justification in many calorimetric studies. This issue is examined in more details in this section.

In the context of heat transfer, one should establish whether the liquid is thermally thin or not, by computing a Biot number. To do so, we first estimate the external heat transfer coefficient by natural convection. In order to get a conservative value, we consider the highest vessel (B) with $H = 237\,\mathrm{mm}$, we assume a mean liquid temperature $T_L = 30\,^\circ\mathrm{C}$ and ambient temperature $T_{\rm amb} = 20\,^\circ\mathrm{C}$. The film temperature is therefore $T_F = 25\,^\circ\mathrm{C}$ so that we take the physical properties at 300 K. After Welty et al. [10], we have $g\beta/\nu^2 = 1.327\times 10^8\,\mathrm{K^{-1}\,m^{-3}}$, where g is gravity acceleration, β the isobaric thermal dilatation coefficient of air, and ν its kinematic viscosity, and the Prandtl number of air is $\mathrm{Pr} = 0.708$. We can compute the Grashof and Rayleigh numbers:

$$\mathrm{Gr} = \frac{g\beta(T_L - T_{\rm amb})H^3}{\nu^2} = 1.85\times 10^7\,, \qquad \mathrm{Ra} = \mathrm{Gr}\,\mathrm{Pr} = 1.31\times 10^7\,.$$

Using the correlation of Churchill & Chu [11], we get the Nusselt number

$$\mathrm{Nu} = \left(0.825 + \frac{0.387\,\mathrm{Ra}^{1/6}}{\left[1+(0.492/\mathrm{Pr})^{9/16}\right]^{8/27}}\right)^2 = 33.7\,.$$

The natural convection heat transfer coefficient is therefore:

$$h_{\rm ext} = \mathrm{Nu}\frac{\lambda_{\rm air}}{H} = 3.68\,\mathrm{W\,m^{-2}\,K^{-1}}\,,$$

with $\lambda_{\rm air} = 2.624\times 10^{-2}\,\mathrm{W\,m^{-1}\,K^{-1}}$. Before computing the Biot number, it should be checked that the thermal resistance $\mathcal{R}_{\rm conv,ext}$ associated with $h_{\rm ext}$ is much larger than the conductive resistance $\mathcal{R}_{\rm cond,glass}$ in glass walls. Taking $\lambda_{\rm glass} = 1\,\mathrm{W\,m^{-1}\,K^{-1}}$ for glass and the widest vessel $d = 2.5\,\mathrm{mm}$ we get

$$\frac{\mathcal{R}_{\rm conv,ext}}{\mathcal{R}_{\rm cond,glass}} = \frac{d/\lambda_{\rm glass}}{1/h_{\rm ext}} = 9.2\times 10^{-3} \ll 1$$

We can now compute the Biot number for the reactor:

$$\mathrm{Bi} = \frac{h_{\rm ext}D_{\rm int}/4}{\lambda_L}$$

where $D_{\rm int}$ is the internal diameter of the vessel and λ_L the conductivity of the liquid. Taking the conservative value $D_{\rm int} = 132\,\mathrm{mm}$ for the widest vessel and $\lambda_L = 0.615\,\mathrm{W\,m^{-1}\,K^{-1}}$, we get $\mathrm{Bi} = 0.2$. It is small, although not sufficiently $\ll 1$ to fully ensure spatial uniformity of the temperature field.

It should be noted that the above calculation assumes that heat transfer would occur by pure conduction in water. In fact, heat transfer in the liquid may be enhanced by cavitation bubbles microstreaming, and also turbulent acoustic streaming [12, 13]. In order to get a numerical confirmation of the latter assertion, we computed the turbulent streaming velocity field computed with the method proposed in Ref. [13], coupled with the unsteady heat equation, where the local heat source is defined as an output of the computed acoustic field [14]. Figure 2a displays the temperature field, which can be seen to be almost spatially uniform. The time-temperature curves displayed Fig. 2b are evaluated at three different points in the liquid

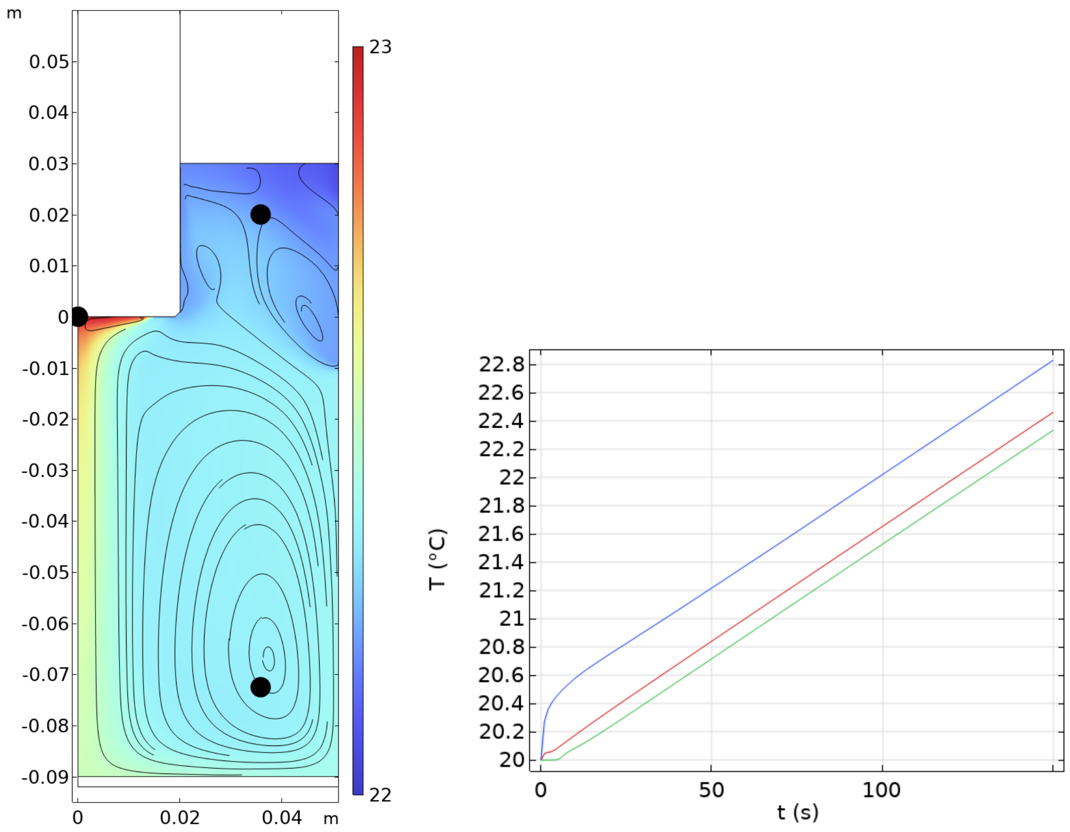

(a) Temperature field (b) Temperature vs. time

Figure 2: (a) Temperature field computed by simulation of the turbulent flow induced by acoustic streaming, coupled with the heat equation. The black lines are the streamlines of the acoustic streaming velocity field (b) Temperature evolution at the three black points materialized in (a): the red line is the temperature at the point below the sonotrode, the green line is the temperature at the point below the free surface, and the red line is the temperature at the deep off-axis point.

(materialized by black dots on Fig. 2a). It can be seen that the curves are almost linear during the full calorimetry experiment, and that their slopes are almost independent of the probe location.

In addition to the above theoretical argumentation, and following the advice of an anonymous referee, we performed additional measurements in two configurations with a 2 L vessel similar to vessel B, varying the immersion depth of the temperature probe from 12 cm down to 1.5 cm, and with an horizontal location halfway between the sonotrode lateral area and the vessel internal wall. It was found that for an immersion depth down to 3.5 cm, no significant change in the value of $\mathcal{P}_{\mathrm{cal}}$ was recorded. This suggests that our calorimetric measurements with an immersion depth of 4 cm systematically used would yield a representative result.

However for an immersion depth of 1.5 cm of the temperature probe, a significantly lower value of $\mathcal{P}_{\mathrm{cal}}$ was measured. This may be a problem for the experiments with a liquid level lower than 4 cm in which case, as mentioned in the main text, the temperature probe was immersed roughly halfway between the vessel bottom and the free surface. This concerns only the two leftmost experiments of Fig. 5 (vessel A) and the leftmost experiment of fig. 7 (vessel C). Despite no firm conclusion can be drawn about the latter, the corresponding points in Figs. 8(a) and 8(c) of our precedent paper [6] do not seem to deviate significantly from the general tendency.

References

[1] H. N. McMurray & B. P. Wilson. “Mechanistic and Spatial Study of Ultrasonically Induced Luminol Chemiluminescence”. In: *The Journal of Physical Chemistry A* 103.20 (May 1999), pp. 3955–3962. ISSN: 1089-5639.

[2] L. Erdey, I. Buzás & K. Vigh. “Luminol as a Fluorescent Acid-Base Indicator”. In: *Talanta. Proceedings of the Dutch Archaeological and Historical Society* 13.3 (Mar. 1966), pp. 463–469. ISSN: 0039-9140.

[3] J. Lind, G. Merenyi & T. E. Eriksen. “Chemiluminescence Mechanism of Cyclic Hydrazides Such as Luminol in Aqueous Solutions”. In: *Journal of The American Chemical Society* 105.26 (Dec. 1983), pp. 7655–7661. ISSN: 0002-7863.

[4] Y. Asakura, T. Nishida, T. Matsuoka & S. Koda. “Effects of Ultrasonic Frequency and Liquid Height on Sonochemical Efficiency of Large-Scale Sonochemical Reactors”. In: *Ultrasonics Sonochemistry* 15.3 (Mar. 2008), pp. 244–250. ISSN: 1350-4177.

[5] Y. Son, M. Lim, M. Ashokkumar & J. Khim. “Geometric Optimization of Sonoreactors for the Enhancement of Sonochemical Activity”. In: *Journal of Physical Chemistry C* 115.10 (2011), pp. 4096–4103.

[6] I. K. Garcia-Vargas, L. Barthe & O. Louisnard. “Extensive investigation of geometric effects in sonoreactors: Analysis by luminol mapping and comparison with numerical predictions.” In: *Ultrasonics Sonochemistry* 99 (2023), p. 106542. ISSN: 1350-4177.

[7] M. Niccoli. *Perceptually improved colormaps. MATLAB Central File Exchange.* https://www.mathworks.com/matlabcentral/fileexchange/28982-perceptually-improved-colormaps. [Online; Retrieved May 11, 2023]. 2023.

[8] D. Borland & R. M. Taylor II. “Rainbow color map (still) considered harmful”. In: *IEEE Computer Graphics and Applications* 27.2 (2007), pp. 14–17.

[9] S. Zeller & D. Rogers. “Visualizing science: How color determines what we see”. In: *Eos, Transactions American Geophysical Union* 101 (2020).

[10] J. Welty, C. E. Wicks, R. E. Wilson & G. L. Rorrer. *Fundamentals of momentum, heat, and mass transfer*. 5th ed. John Wiley & Sons, 2007. ISBN: 978-0470128688.

[11] S. W. Churchill & H. H. S. Chu. “Correlating equations for laminar and turbulent free convection from a vertical plate”. In: *International Journal of Heat and Mass Transfer* 18.11 (1975), pp. 1323–1329. ISSN: 0017-9310.

[12] T. Nowak, C. Cairós, E. Batyrshin & R. Mettin. “Acoustic streaming and bubble translation at a cavitating ultrasonic horn”. In: *Recent developments in nonlinear acoustics: 20th International Symposium on Nonlinear Acoustics including the 2nd International Sonic Boom Forum*. Vol. 1685. AIP Publishing. 2015, p. 020002.

[13] O. Louisnard. “A viable method to predict acoustic streaming in presence of cavitation”. In: *Ultrasonics sonochemistry* 35 (2017), pp. 518–524.

[14] Q. Goris, A. Bampouli, M. N. Hussain, O. Louisnard, G. D. Stefanidis & T. Van Gerven. “A new strategy for modelling sonochemical reactors: Coupling of the non-linear Louisnard model with mass and heat transport equations with applications to cavitating viscous fluids”. In: *Ultrasonics Sonochemistry* 112 (2025), p. 107114.
